# Radioimmunotheranostic Pair Based on the Anti-HER2 Monoclonal Antibody: Influence of Chelating Agents and Radionuclides on Biological Properties

**DOI:** 10.3390/pharmaceutics13070971

**Published:** 2021-06-27

**Authors:** Ana Cláudia Camargo Miranda, Sofia Nascimento dos Santos, Leonardo Lima Fuscaldi, Luiza Mascarenhas Balieiro, Maria Helena Bellini, Maria Inês Calil Cury Guimarães, Elaine Bortoleti de Araújo

**Affiliations:** 1 Hospital Israelita Albert Einstein, Instituto Israelita de Ensino e Pesquisa, Sao Paulo 05652-900, Brazil; 2 Instituto de Pesquisas Energéticas e Nucleares, IPEN/CNEN, Sao Paulo 05508-000, Brazil; snsantos@usp.br (S.N.d.S.); luia_m@uol.com.br (L.M.B.); mbmarumo@ipen.br (M.H.B.); ebaraujo@ipen.br (E.B.d.A.); 3 Departamento de Ciências Fisiológicas, Faculdade de Ciências Médicas da Santa Casa de São Paulo, Sao Paulo 01221-020, Brazil; leonardo.fuscaldi@hotmail.com; 4 Instituto de Radiologia do Hospital das Clínicas da Faculdade de Medicina da Universidade de São Paulo, Sao Paulo 05403-911, Brazil; micguima@usp.br

**Keywords:** HER2 oncogene, breast cancer, [^111^In]In-DTPA-trastuzumab, [^177^Lu]Lu-DOTA-trastuzumab, radioimmunoconjugate, radioimmunotheranostic pair

## Abstract

The oncogene HER2 is an important molecular target in oncology because it is associated with aggressive disease and the worst prognosis. The development of non-invasive imaging techniques and target therapies using monoclonal antibodies is a rapidly developing field. Thus, this work proposes the study of the radioimmunotheranostic pair, [^111^In]In-DTPA-trastuzumab and [^177^Lu]Lu-DOTA-trastuzumab, evaluating the influence of the chelating agents and radionuclides on the biological properties of the radioimmunoconjugates (RICs). The trastuzumab was immunoconjugated with the chelators DTPA and DOTA and radiolabeled with [^111^In]InCl_3_ and [^177^Lu]LuCl_3_, respectively. The stability of the RICs was evaluated in serum, and the immunoreactive and internalization fractions were determined in SK-BR-3 breast cancer cells. The in vivo pharmacokinetics and dosimetry quantification and the ex vivo biodistribution were performed in normal and SK-BR-3 tumor-bearing mice. The data showed that there was no influence of the chelating agents and radionuclides on the immunoreactive and internalization fractions of RICs. In contrast, they influenced the stability of RICs in serum, as well as the pharmacokinetics, dosimetry and biodistribution profiles. Therefore, the results showed that the nature of the chelating agent and radionuclide could influence the biological properties of the radioimmunotheranostic pair.

## 1. Introduction

Personalized oncology is based on evidence and offers more assertive decisions for each patient, leading to successful results and reduced healthcare costs. It involves genomic analysis, target-specific drugs, treatment and diagnosis by molecular imaging. It is a promising new approach with remarkable impact on personalized medicine [1]. HER2 is an important molecular target in oncology because it is related to more aggressive tumor development in patients [2,3,4]. Breast cancer is a tumor type that overexpresses the HER2 oncogene in approximately 20% to 30% of cases; this expression is associated with the worst prognosis [3,5,6]. Currently, the available diagnostic methods are invasive and may present inconsistent results due to intratumoral heterogeneity, and the sample size of the biopsied tumor may not represent the whole tumor expression, as well as the possibility of promoting metastatic lesions by repeated biopsies [2].

In this sense, innovative alternatives have been widely investigated and the use of radiolabeled monoclonal antibodies is a rapidly developing field, aiming to find new agents for radioimmunodiagnosis (RID) and radioimmunotherapy (RIT) [3,4]. The RID is a non-invasive imaging technique, which offers advantages in the diagnosis and staging of HER2-positive tumors, allowing the selection of patients who are responsive to a targeted therapy, as well as allowing the monitoring the therapeutic response and the identification of patients who become resistant to immunotherapy [2]. On the other hand, RIT allows for combining specific molecular target therapy with systemic treatment, in order to increase the cytotoxic effect of antibodies by means of their association with a radionuclide, providing the radiation deposition at the site of interest without compromising healthy tissues [7,8,9,10,11]. In addition, there is a great deal of interest in the development of radioimmnunoconjugates (RICs) for theranostic purposes [7].

The rapid growth in the number of molecular biomarkers and the development of drugs for targeted therapy in the treatment of breast cancer began after the discovery of tyrosine kinase receptors, which allowed the emergence of the first target therapy using the humanized anti-HER2 IgG1 monoclonal antibody, trastuzumab, approved by the Food and Drug Administration (FDA) in 1998 [12,13,14]. Due to its importance, trastuzumab has been radiolabeled and evaluated for the purposes of radioimmunotheranostics. In particular, the radioimmunotheranostic pair allows the diagnosis and staging of the disease, the confirmation of the target expression and, consequently, the choice of the patient for specific treatment, which is in agreement with personalized medicine [3,5,6].

In this context, the choice of the radionuclide depends on its physical properties, considering the slow blood clearance of trastuzumab, its local availability and economic viability for routine use [3,7]. On the other hand, the coordination chemistry of each metallic or lanthanide radionuclide requires a specific chelating agent, which can directly influence the biological properties of the RIC [12,15,16,17,18,19,20]. Therefore, the study of the influence of different bifunctional chelating agents and radionuclides on the biological properties of RICs represents an important field of investigation for the development of new theranostic agents [21].

In this work, the trivalent metal ions indium-111 (^111^In) and lutetium-177 (^177^Lu) were chosen for radiolabeling the trastuzumab antibody. ^111^In is produced in a cyclotron by the nuclear reaction ^111^Cd (*p*, *n*) ^111^In, and it has a half-life of 67.9 h (2.80 d). It decays 100% by electron capture with gamma (γ) ray emissions [171 KeV (91%) and 245 KeV (94%)]. It is used for diagnosis in single photon emission computed tomography (SPECT) imaging [17]. S-2-(4-isothiocyanatobenzyl)-diethylenetriamine pentaacetic acid (p-SCN-Bn-DTPA) was conjugated to the antibody because it is an acyclic chelating agent that has the advantage of allowing high labeling efficiency with ^111^In, leading to greater thermodynamic stability compared to other chelators [22]. On the other hand, ^177^Lu can be produced in a reactor by irradiating the enriched ^176^Lu (^176^Lu (*n*, γ) ^177^Lu) for a low-cost, high-yield and medium-specific activity. It decays to hafnium-177 (^177^Hf) by beta (β^−^) emissions [177 KeV (12%), 385 KeV (9%) and 498 KeV (79%)] and low-abundance γ ray emissions [113 KeV (7%) and 208 KeV (11%)], with a half-life of 160.4 h (6.7 d) [17]. Additionally, 1,4,7,10-tetraazacyclododecane-1,4,7,10-tetraacetic acid mono-N-hydroxysuccinimide ester (DOTA-NHS-ester) was conjugated to trastuzumab because it is a macrocyclic chelating agent that promotes greater kinetic and thermodynamic stabilities when radiolabeled with ^177^Lu [22]. The γ emissions of ^177^Lu allow it to be used for RID, particularly to track the uptake of lesions after the therapeutic procedure. Regarding RIT, among the particle-emitting radionuclides, ^177^Lu has adequate physical properties that lead to a safer treatment, such as low-energy β^−^ emission which has a short range (0.2–0.3 mm), promoting a lower radiation dose to the bone marrow compared, for example, to yttrium-90 (^90^Y). Furthermore, the cross-fire effect of β^−^ may be more effective than alpha (α) particles emitted by radium-223 (^223^Ra), bismuth-213 (^213^Bi) and astatine-211 (^211^At) [7,8,23]. In cancer treatment, the antibody interacts with a limited number of cells in the tumor mass. However, if a radiolabeled antibody is used, more cells will be killed due to the cross-fire of the emitted particles (β^−^, α and Auger electrons) [24].

Thus, this work aimed to study the potential of the [^111^In]In-DTPA-trastuzumab and [^177^Lu]Lu-DOTA-trastuzumab as a radioimmunotheranostic pair, evaluating the influence of different chelating agents and radionuclides on the biological properties of trastuzumab.

## 2. Materials and Methods

### 2.1. Immunoconjugation and Radioimmunoconjugation 

**Figure 1:**
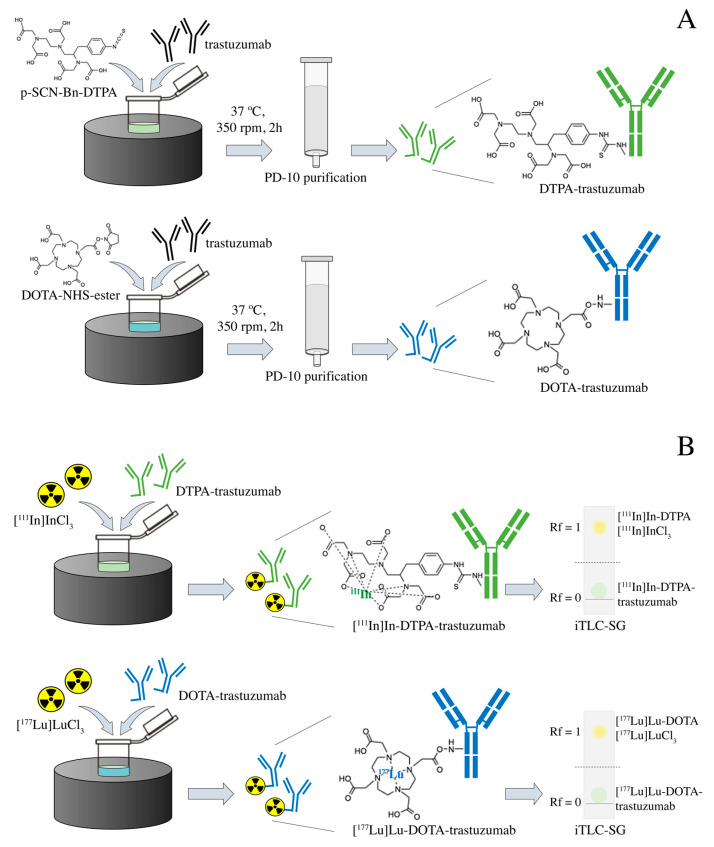
Scheme of the (**A**) immunoconjugation with DTPA and DOTA and (**B**) radiolabeling with [^111^In]InCl_3_ and [^177^Lu]LuCl_3_ of trastuzumab.

Immunoconjugation and radioimmunoconjugation were performed as previously described [25]. Briefly, the trastuzumab (Herceptin^®^, Roche, Basileia, Switzerland), at an initial concentration of 10.5 mg·mL^−1^, was previously purified by centrifugation using the concentrator tube (Amicon^®^ Ultra 10,000 MWCO, Merck Millipore, Burlington, MA, USA) with 0.2 M sodium bicarbonate buffer (pH 8.5) at 3000× *g*/4 °C. Conjugation to the bifunctional chelating agents p-SCN-Bn-DTPA and DOTA-NHS-ester (Macrocyclics, Plano, TX, USA) in a 1:20 (antibody:chelator) molar ratio, were performed by incubation at 37 °C and 350 rpm for 2 h. The volume of the immunoconjugation reaction was 1.5 mL. The immunoconjugates were purified through a molecular exclusion column (Sephadex—G25 PD-10, GE Healthcare, Bronx, NY, USA), using a 0.25 M ammonium acetate buffer (pH 6.5)—Figure 1A. The immunoconjugates DTPA-trastuzumab and DOTA-trastuzumab were radiolabeled, respectively, with carrier-free [^111^In]InCl_3_ (Curium, London, United Kingdom) and [^177^Lu]LuCl_3_ (I.D.B. Holland B.V., Baarle-Nassau, Netherlands), ≥500 GBq/mg, with molar activity of 43.09 MBq/nmol at 42 °C and 450 rpm for 1 h (pH 6.5)—Figure 1B.

Radiochemical purity was assessed by ascending chromatography, using iTLC-SG (Agilent Technologies, Santa Clara, CA, USA) and 0.1 M sodium citrate buffer (pH 5.0) as eluent (Figure 1B). When the radiochemical purity was <90%, the RIC was purified through a molecular exclusion column (Sephadex^®^—G25 PD-10, GE Healthcare, Bronx, NY, USA), using 0.25 M ammonium acetate buffer (pH 6.5). High-purity-grade reagents were purchased from Merck Millipore (Burlington, MA, USA).

### 2.2. In Vitro Studies

#### 2.2.1. Evaluation of the Stability of the Radioimmunoconjugates in Serum

The stability of [^111^In]In-DTPA-trastuzumab and [^177^Lu]Lu-DOTA-trastuzumab was evaluated in serum. An aliquot of each RIC (50 µL; ≈9 MBq) was incubated with 450 µL of serum (37 °C; 450 rpm) for 4, 24, 48, 72, 96, 120, 144 and 168 h. For each time-point, an aliquot was analyzed by ascending chromatography as previously described.

#### 2.2.2. Cell Culture

The SK-BR-3 (ATCC HTB-30TM) cell line was grown in DMEM medium supplemented with 10% fetal bovine serum and 50 µg/mL gentamicin (Gibco, Life Technologies, Austin, TX, USA). The cells were kept in humidified air containing 5% CO_2_ at 37 °C. The cells were grown to confluence and then harvested via trypsinization. After centrifugation (1200 rpm; 5 min), the cells were resuspended in supplemented DMEM medium for in vitro studies or in matrigel/supplemented DMEM medium (1:1) for in vivo tumor development.

#### 2.2.3. Determination of the Immunoreactive Fraction 

The determination of the immunoreactive fraction of each RIC was performed according to Lindmo and coworkers [26].

The SK-BR-3 cells suspension was serially diluted: 5, 2.5, 1.25, 0.625, 0.312 and 0.156 × 10^6^ cells per vial. For specific binding (SB), 1% BSA in PBS and 2.2 pmol of the RIC were added to each vial (*n* = 3). For non-specific binding (NSB), 1% BSA in PBS, 2.2 nmol of trastuzumab and 2.2 pmol of the RIC were added to each vial (*n* = 3). After incubation for 1 h (4 °C; gentle agitation), the vials were centrifuged (2000 rpm; 5 min) and the supernatants were collected. The vials containing pellets and supernatants were quantified in an automatic gamma counter.

Data were analyzed by linear regression using the GraphPad Prism v. 8.3.1 software (GraphPad Software Inc.—La Joya, CA, USA). The value in the *Y*-axis, when the value in the *X*-axis is equal to zero, corresponds to the immunoreactive fraction (r) expressed as 1/r.

#### 2.2.4. Internalization

The internalization of RICs into SK-BR-3 cells was evaluated as described in previous studies, with some modifications [27,28]. For each RIC, [^111^In]In-DTPA-trastuzumab and [^177^Lu]Lu-DOTA-trastuzumab, 1% BSA in PBS and 2.2 pmol of the RIC were added to a vial containing 2 × 10^6^ SK-BR-3 cells. After 1, 4 and 24 h of incubation at 37 °C, the vials were centrifuged (2000 rpm; 5 min), the supernatants were discarded, and the cell pellets were resuspended with 500 μL of 0.2 M acetic acid buffer (pH 2.8) and incubated at 4 °C for 10 min, removing the receptor-bound fraction (but not internalizing it). Then, the vials were centrifuged (2000 rpm; 5 min.), and the supernatants were collected. Pellets (the fraction internalized into the cells) and supernatants were quantified in the automatic gamma counter. The result was expressed as a percentage of total activity in each fraction as a function of time.

### 2.3. In Vivo Studies

Inbred female BALB/c and BALB/c nude mice (4–8 weeks; 20–30 g) were supplied by the animal facility of the Centro de Experimentação e Treinamento em Cirurgia (CETEC) of the Hospital Israelita Albert Einstein (Sao Paulo, SP, Brazil), certified by the Association for Assessment and Accreditation of Laboratory Animal Care International (AAALAC). The animals (3–5 mice per cage) were maintained under specific pathogen-free (SPF) conditions with *ad libitum* access to food and water. The mouse room was temperature- (22 ± 3 °C) and humidity (55 ± 10%)-controlled, with filtered air and a regulated light–dark cycle (12/12 h), with lights turned on at 07:00 a.m. Animals received nest material (paper) and rolls as environmental enrichment. All procedures involving mice were conducted in agreement with the National Council for Animal Experimentation Control (CONCEA) and were approved by the Ethics Committee on Animal Use of the Instituto de Pesquisas Energéticas e Nucleares (IPEN)—(protocol *n*° 170/16, 15 April 2016) and the Hospital Israelita Albert Einstein (HIAE*)*—(protocol *n*° 3463/18, 12 July 2018).

#### 2.3.1. Pharmacokinetics 

The pharmacokinetic study was performed in normal female BALB/c mice for both RICs, [^111^In]In-DTPA-trastuzumab and [^177^Lu]Lu-DOTA-trastuzumab. At 0, 4, 24, 48, 72, 96, 144 and 168 h after injection of each RIC, 60 µL of blood was collected from the animal’s orbital plexus after anesthetic induction with 5% isoflurane. The radioactivity of the blood samples was measured in an automatic gamma counter and the pharmacokinetic parameters were quantified after adjustment for a two-compartment distribution model. Blood clearance was calculated by the ratio between the injected activity and the area under the curve (AUC), and the effective half-life was calculated considering the half-life of the slow phase as the biological half-life.

#### 2.3.2. Xenographic Breast Tumor Animal Model

The xenographic breast tumor animal model was developed in female BALB/c nude mice. In each animal, an aliquot (100 µL) containing 5 × 10^6^ SK-BR-3 breast cancer cells in matrigel:DMEM medium (1:1) was subcutaneously inoculated in the right lower flank. Tumor growth was assessed weekly using a caliper rule. The tumor volume was calculated as proposed by Faustino-Rocha and co-workers, considering the measurements obtained from the smallest and largest diameter of the tumor mass [29]. When the average volume of tumor masses reached approximately 196 mm^3^ in diameter, the tumors were used for histopathological analysis or the animals were used in biodistribution studies.

#### 2.3.3. Histopathological Analysis

Tumor tissue was obtained from the mice and fixed in formalin (10% *v*/*v* in PBS). The hematoxylin- and eosin-staining procedure was performed on paraffin-embedded sections (5 µm) mounted on glass slides. The images of histological sections were captured by a light Nikon Eclipse E600 microscope (Tokyo, Japan).

#### 2.3.4. Ex Vivo Biodistribution Studies

The biodistribution studies were conducted in normal female BALB/c mice and in SK-BR-3 tumor-bearing female BALB-c nude mice for both RICs, [^111^In]In-DTPA-trastuzumab and [^177^Lu]Lu-DOTA-trastuzumab. An aliquot of 18.5 MBq of each RIC was intravenously injected into the tail vein of animals after anesthetic induction with 5% of isoflurane. After 4, 24, 48, 72, 96 and 168 h since RIC injection into normal animals and after 24, 72, and 168 h since RIC injection into tumor-bearing mice, animals were euthanized by anesthetic overdose of a combination of ketamine (300 mg/kg) and xylazine (30 mg/kg). The organs and tissues of interest were removed, weighed and quantified in the automatic gamma counter. The percentages of injected dose per gram of tissue (%ID/g) were calculated using a standard dose containing the same amount of the dose injected into the mice and defined as 100%. For tumor-bearing mice, the target-to-non-target ratio was calculated as tumor to contralateral muscle.

#### 2.3.5. Dosimetric Study

The biodistribution data of RICs from six normal mice were used to estimate the absorbed dose in the period of 4, 24, 48, 72, 96 and 168 h, and then the extrapolation for the dose in humans was calculated using the Medical Internal Radiation Dose (MIRD) methodology [30] and the method described by Sparks and Aydogan [31]. The data from the International Commission on Radiological Protection (ICRP-60 and ICRP-89) [32,33] were also used, and the absorbed fractions were obtained from the OLINDA/EXT software [34,35]. The absorptions of the RICs were calculated using the residence times and the cumulative activity integral of the MIRD methodology.

### 2.4. Statistical Analysis

Statistical analysis was performed using the GraphPad Prism v. 8.3.1 software (GraphPad Software Inc.—La Joya, CA, USA). Data were expressed as mean ± error. The means of two groups were compared using the Student’s *t*-test. The means of three or more groups were compared by analysis of variance (ANOVA), followed by Tukey’s multiple comparison test (one-way ANOVA) or the Bonferroni test (two-way ANOVA). *p*-values ≤ 0.05 were considered statistically significant different.

## 3. Results

### 3.1. Immunoconjugation and Radioimmunoconjugation 

The immuno- and radioimmunoconjugation were performed with no further modification from the previous proposed protocol [25], at a 1:20 (antibody:chelator) molar ratio. The RICs presented radiochemical purity >90%; however, for [^177^Lu]Lu-DOTA-trastuzumab, the radiochemical yield was about 84% and, then it was necessary to perform the purification in a molecular exclusion column to achieve radiochemical purity >90%. For [^111^In]In-DTPA-trastuzumab, this step was not necessary.

### 3.2. In Vitro Studies

#### 3.2.1. Evaluation of the Stability of the Radioimmunoconjugates in Serum

**Figure 2:**
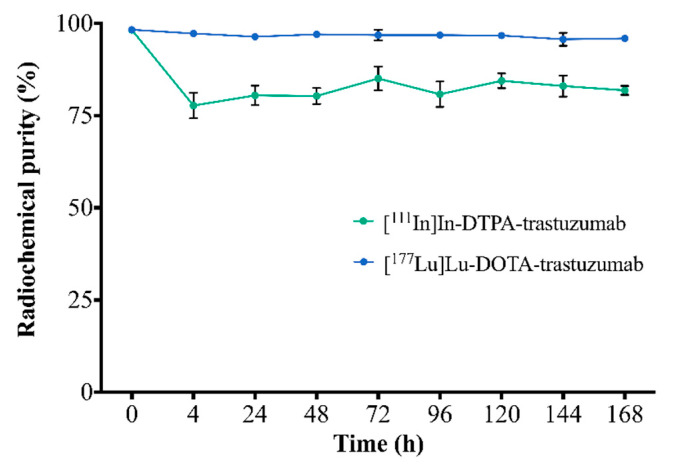
Assessment of the stability of RICs in serum by ascending chromatography. Values are expressed as mean ± SD (*n* = 6).

The stability of RICs in serum, assessed by ascending chromatography, is shown in Figure 2. [^111^In]In-DTPA-trastuzumab and [^177^Lu]Lu-DOTA-trastuzumab showed radiochemical purity >75% and 94%, respectively, up to 168 h.

#### 3.2.2. Determination of the Immunoreactive Fraction 

**Figure 3:**
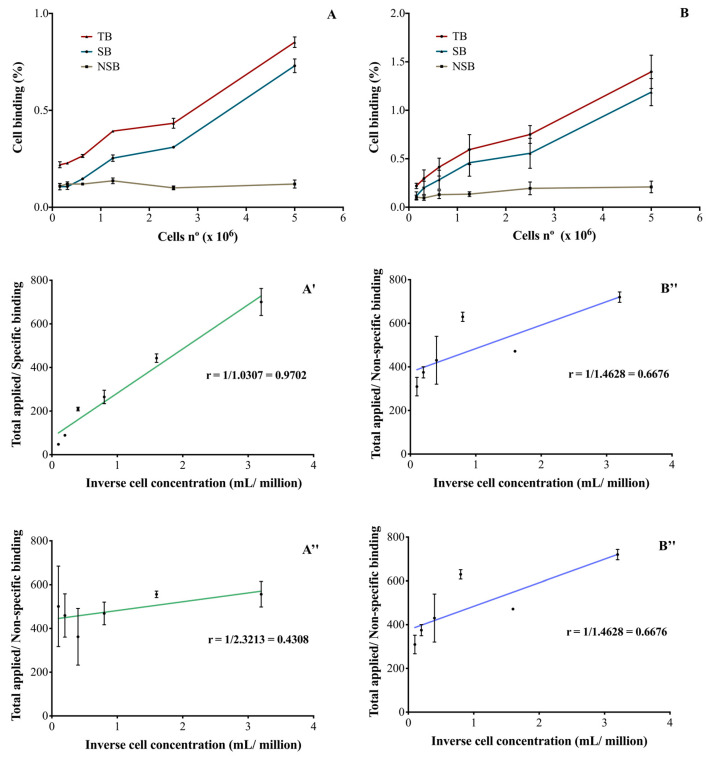
Percentage of RICs binding to SK-BR-3 cells (HER2-positive) of (**A**) [^111^In]In-DTPA-trastuzumab and (**B**) [^177^Lu]Lu-DOTA-trastuzumab and the evaluation of the immunoreactive fraction of the RICs: (**A′**) and (**B′**) specific binding to SK-BR-3 cells and (**A″**) and (**B″**) non-specific binding. TB—total binding; SB—specific binding; NSB—non-specific binding. The values are expressed as mean ± SEM (*n* = 3).

After immuno- and radioimmunoconjugation, both RICs were able to bind SK-BR-3 cells in a cellular-concentration-dependent manner (Figure 3A,B). The binding was specific and the unlabeled monoclonal antibody was able to inhibit RICs binding to cells. In the described conditions, it was not possible to obtain a saturation of the receptors and therefore, the Kd and Bmax parameters were not calculated. The immunoreactive fractions of [^111^In]In-DTPA-trastuzumab (Figure 3A′,A″) and [^177^Lu]Lu-DOTA-trastuzumab (Figure 3B′,B″) were determined by plotting the double inverse plot of the applied radiolabeled antibody over the specific (Figure 3A′,B′) and non-specific (Figure 3A″,B″) binding as a function of the inverse cell concentration. Theoretically, it was assumed that the unconjugated and unlabeled trastuzumab has an immunoreactivity of 100% [26].

For [^111^In]In-DTPA-trastuzumab (Figure 3A′) and [^177^Lu]Lu-DOTA-trastuzumab (Figure 3B′), 97 and 98% of immunoreactivity was preserved, respectively. On the other hand, in the presence of the competitor (unlabeled trastuzumab), a decrease in the immunoreactivity percentages was observed for [^111^In]In-DTPA-trastuzumab (43%—Figure 3A″) and [^177^Lu]Lu-DOTA-trastuzumab (66%—Figure 3B″).

#### 3.2.3. Internalization

**Figure 4:**
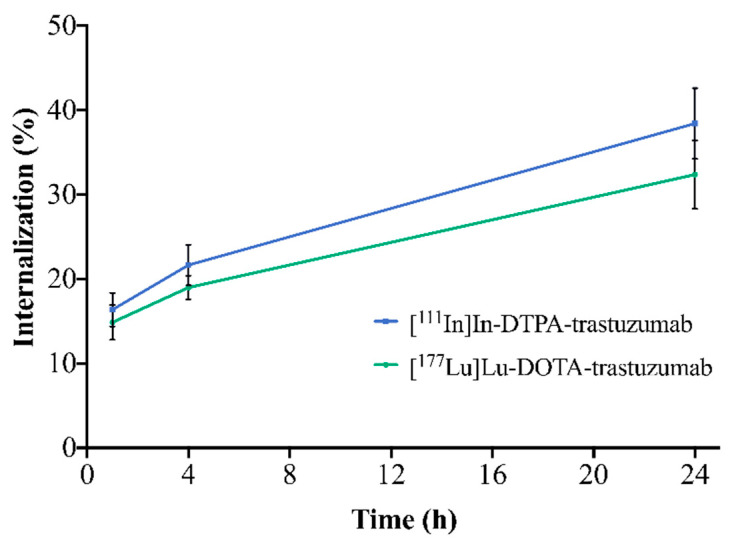
Internalization of [^111^In]In-DTPA-trastuzumab and [^177^Lu]Lu-DOTA-trastuzumab into SK-BR-3 cells after 1, 4 and 24 h of incubation. Values are expressed as mean ± SD (*n* = 4).

The internalization of the RICs into SK-BR-3 cells is illustrated in Figure 4. For both RICs, the percentage of internalization increased with time. [^111^In]In-DTPA-trastuzumab showed internalization of 16.4% (1 h), 21.6% (4 h) and 38.4% (24 h). [^177^Lu]Lu-DOTA-trastuzumab presented 14.9% (1 h), 19.0% (4 h),and 32.4% (24 h) of internalization. However, no significant differences between RICs were observed when comparing the same evaluated times (*p* > 0.05).

#### 3.2.4. Pharmacokinetics

**Figure 5:**
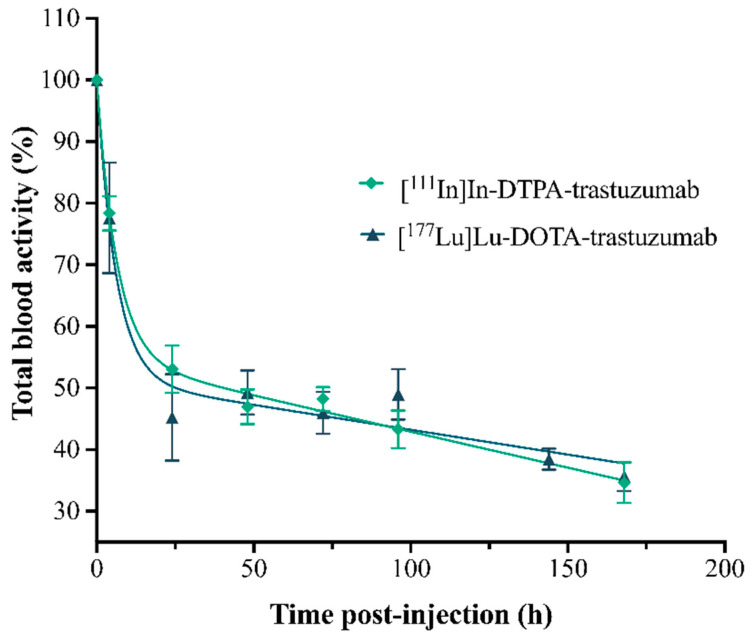
Percentage curves of the activities of the RICs, [^111^In]In-DTPA-trastuzumab and [^177^Lu]Lu-DOTA-trastuzumab, in total blood of normal female BALC/c mice. Values are expressed as mean ± SEM (*n* = 4–5).

The pharmacokinetic parameters, by two-compartment mathematical models, were obtained from a plasma concentration curve (% of RIC’s activity in total blood) as a function of time (Figure 5), simulating the processes of absorption, distribution, metabolism and excretion (ADME) of the RICs (Table 1).

#### 3.2.5. Xenographic Breast Tumor Animal Model and Histopathological Analysis

**Figure 6:**
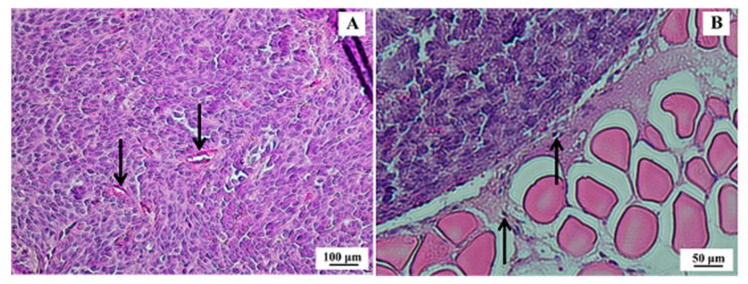
Histological section of SK-BR-3 breast tumor. (**A**) Presence of a dense tumor mass indicating high mitotic activity and cellular pleomorphism with some cuboid cells; however, with a higher frequency of spindle cells and scarce basophilic cytoplasm (100× magnification)—arrows indicate tumor vessels. (**B**) Border region of the tumor (strongly acidophilic cell mass) and muscle tissue (200× magnification)—arrows indicate tumor cells migrating to muscle tissue. Hematoxylin & eosin (H&E) staining.

After 21 days of inoculation of SK-BR-3 cells, the tumors presented an average volume of 195.8 ± 66.3 mm^3^. Histopathological analysis confirmed morphological features compatible with human SK-BR-3 breast tumors (Figure 6).

### 3.3. Ex Vivo Biodistribution Studies

**Figure 7:**
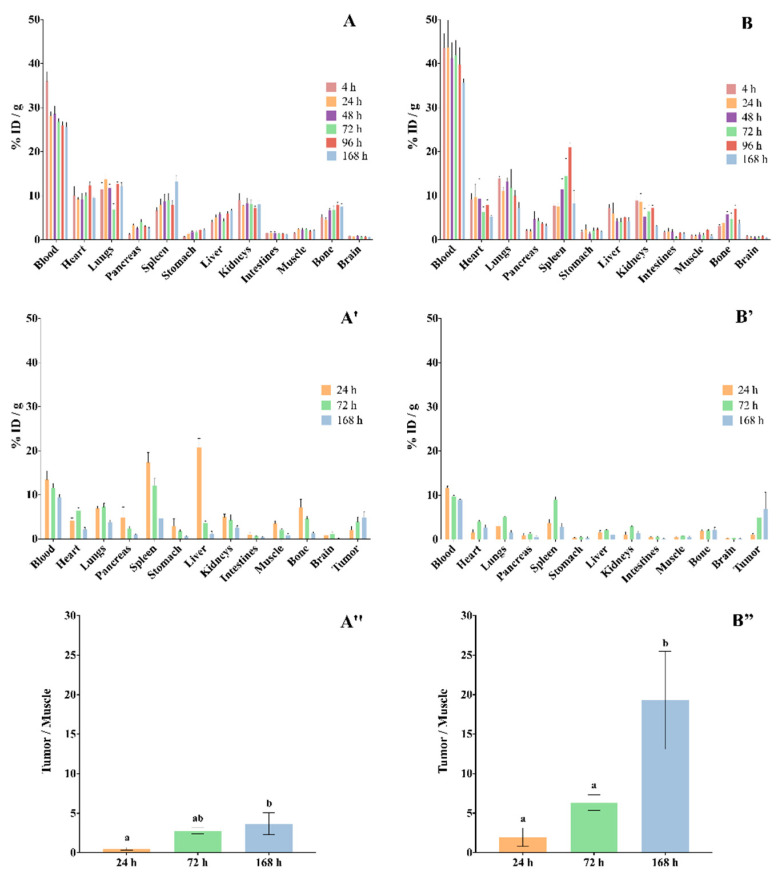
Biodistribution of (**A**) [^111^In]In-DTPA-trastuzumab and (**B**) [^177^Lu]Lu-DOTA-trastuzumab in mice. (**A**,**B**) normal female BALB/c mice. (**A′**,**B′**) SK-BR-3 breast-tumor-bearing female BALC/c nude mice. (**A″**,**B″**) Target-to-non-target ratio (tumor/muscle). Values are expressed as mean ± SEM (*n* = 4–5). Different letters indicate significant differences (*p* < 0.05). ID—injected dose.

The ex vivo biodistribution of RICs, [^111^In]In-DTPA-trastuzumab and [^177^Lu]Lu-DOTA-trastuzumab, in normal female BALB/c mice are shown in Figure 7A,B, and in SK-BR-3 breast-tumor-bearing female BALC/c nude mice in Figure 7A′,B.

The data showed that both RICs presented slow blood clearance, associated with significative uptake by the liver, spleen and kidneys. The RICs also accumulated in the heart, lungs, intestines and bones. The uptake of [^177^Lu]Lu-DOTA-trastuzumab by bone was lower than that of [^111^In]In-DTPA-trastuzumab at 4 h and 168 h (*p* < 0.05). On the other hand, the brain, pancreas, stomach and muscle presented low accumulation of RICs.

Ex vivo biodistribution data that were obtained in xenograft breast-tumor-bearing mice showed that tumor uptake was significant and increased over the evaluated period for both RICs (Figure 7A″,B″), [^111^In]In-DTPA-trastuzumab [%ID/g = 2.02% ± 1.49 (24 h); 3.88% ± 1.55 (72 h); 4.92% ± 1.18 (168 h)] and [^177^Lu]Lu-DOTA-trastuzumab [%ID/g = 1.02% ± 0.46 (24 h); 4.97% ± 1.34 (72 h); 6.94% ± 7.43 (168 h)]. Furthermore, the target-to-non-target ratio (tumor/muscle) increased within time for both RICs. However, the ratios of [^177^Lu]Lu-DOTA-trastuzumab were higher than those of [^111^In]In-DTPA-trastuzumab.

#### Dosimetric Study

The dosimetric data of [^111^In]In-DTPA-trastuzumab and [^177^Lu]Lu-DOTA-trastuzumab for the four organs of greatest absorption and interest are summarized in Table 2.

The data are extrapolated for humans using the diagnostic dose for [^111^In]In-DTPA-trastuzumab and the therapeutic dose for [^177^Lu]Lu-DOTA-trastuzumab.

## 4. Discussion

The nature of the chelating agent and radionuclide influences the biological properties of RICs. In this sense, in the development of a monoclonal antibody-based radioimmunotheranostic pair, it is important to evaluate such influence. In the present work, the anti-HER2 monoclonal antibody trastuzumab was immuno- and radioimmunoconjugated with [^111^In]In-DTPA and [^177^Lu]Lu-DOTA. The radioimmunotheranostic pair, [^111^In]In-DTPA-trastuzumab and [^177^Lu]Lu-DOTA-trastuzumab, were compared concerning their immunoreactivity and biological properties in order to assess the influence of the chelating agents and radionuclides.

The number of chelators per antibody molecule may influence the radiochemical stability of the radioimmunoconjugate. Previous MALDI–TOF studies revealed an average number of 6–7 molecules of p-SCN-Bn-DTPA and 8–9 molecules of DOTA-NHS-ester coupled to trastuzumab for a 1:20 M ratio [25]. The [^177^Lu]Lu-DOTA immunoconjugate showed greater stability in serum than [^111^In]In-DTPA, especially in the first 4 h of incubation. Indium atoms present similar coordination chemistry and biological properties compared to Fe^3+^. Then, in vivo, a slight transchelation process of ^111^In may occur in the presence of specific protein-binding sites, such as transferrin, lactoferrin and ferritin [22,36]. Lub and coworkers observed a reduction of 7% per day in the stability of [^111^In]In-DTPA-trastuzumab after incubation in serum at 37 °C, related to the transchelation of ^111^In to transferrin [37]. In the same direction, Blend and coworkers reported a slight decrease in stability in human plasma after 96 h of incubation [38]. In biodistribution studies, the bone uptake of [^111^In]In-DTPA-trastuzumab increased with time and this uptake is probably related to the free ^111^In. On the other hand, [^177^Lu]Lu-DOTA-trastuzumab showed no stability reduction in serum within time (*p* > 0.05), which is consistent with the study of Rasaneh and coworkers that showed stability for up to 96 h [8]. Previously, our research group showed stability of both RICs, [^111^In]In-DTPA-trastuzumab and [^177^Lu]Lu-DOTA-trastuzumab, in saline and under refrigeration up to 168 h, with radiochemical purity >96% [25]. Bone uptake of [^177^Lu]Lu-DOTA-trastuzumab was smaller than that of [^111^In]In-DTPA-trastuzumab at 4 h and 168 h (*p* < 0,05), such uptake being attributable to the free isotope in circulation.

The incorporation of [^111^In]In-DTPA and [^177^Lu]Lu-DOTA to the trastuzumab molecule did not compromise the immunoreactivity of the monoclonal antibody. The results showed 97 and 98% of the preserved immunoreactivity for the [^111^In]In-DTPA-trastuzumab and [^177^Lu]Lu-DOTA-trastuzumab, respectively. Incubation with an excess of unlabeled trastuzumab reduced the binding of RICs to SK-BR-3 cells, indicating the binding specificity. The percentages of the immunoreactive fraction obtained for both RICs in this study were higher than those previously reported [8,23,27,37]. The immunoreactivity of a RIC is related to the antibody:chelator molar ratio employed in the immunoconjugation, the possible binding of the chelator to the antigen binding site and the type of chelator [39,40,41,42]. In this study, there was no influence of the different chelating agents (DTPA and DOTA) and radionuclides (^111^In and ^177^Lu) on the immunoreactivity of the monoclonal antibody trastuzumab.

The incorporation of [^111^In]In-DTPA and [^177^Lu]Lu-DOTA also did not influence the internalization of the trastuzumab into the SK-BR-3 cells. Both RICs showed an increment of internalization within the same time-frame, and no significant differences were observed between them. The internalization process is slow and probably related to the binding of RICs to the HER2 receptors expressed on the surface of SK-BR-3 cells. The slow internalization is possibly justified by the molecular weight of trastuzumab (≈150 kDa). After internalization, the antibody is metabolized intracellularly to smaller peptides and amino acids [43]. The percentage of internalization obtained in this study, for both RICs, was higher than was previously published [27,37]. These differences are probably due to the different molar ratios (antibody:chelator) and cell lines used by each author.

The pharmacokinetic study showed that both RICs presented slow clearance, influencing the slow phase half-life (elimination)—T½ Ke. The clearance of [^177^Lu]Lu-DOTA-trastuzumab (0.04 mL·h^−1^) was slower than that of [^111^In]In-DTPA-trastuzumab (0.28 mL·h^−1^). Antibodies commonly present slow clearance [44,45]. In addition, RICs showed a low volume of distribution (Vd), indicating that they remain longer in circulation and distribute slowly to the tissues. [^177^Lu]Lu-DOTA-trastuzumab showed lower Vd (0.001 L) compared to [^111^In]In-DTPA-trastuzumab (0.005 L), which is in agreement with clearance data. Therefore, [^177^Lu]Lu-DOTA-trastuzumab presented an effective half-life (T½ ef) of 144.78 h, which was double that found for [^111^In]In-DTPA-trastuzumab (62.42 h). Trastuzumab has non-linear pharmacokinetics, and its renal excretion is very small [43,46]. This clearance pattern is due to the high molecular weight of trastuzumab.

The biodistribution data showed high uptake of RICs by liver, spleen and kidneys. This associated uptake indicates that RICs are metabolized by the liver and spleen, and that the metabolic elimination occurs through the kidneys [47,48,49]. Furthermore, biodistribution showed slow blood clearance, which is in agreement with the pharmacokinetic data and with the previously determined partition coefficients, [^111^In]In-DTPA-trastuzumab (log *p* ≈ 3.0) and [^177^Lu]Lu-DOTA-trastuzumab (log *p* ≈ 1.8), that showed lipophilic features of both RICs [25]. Cooper and coworkers obtained similar results for the rituximab antibody immunoconjugated to different chelators, including DTPA and macrocyclic chelators [50].

The uptake of RICs by the lungs and intestines was also observed, which may be associated with the physiological presence of the HER2 oncogene in these organs. [27,37] On the other hand, low uptake by the brain, pancreas, stomach and muscle was observed. The uptake of [^177^Lu]Lu-DOTA-trastuzumab by bone was lower than that of [^111^In]In-DTPA-trastuzumab. The metal complexes of the DOTA conjugates exhibit greater in vivo stability compared to the complexes of the chelating agent DTPA [51]. This profile is in agreement with Lub de Hooge and coworkers, who also demonstrated low bone uptake resulting from the possible decomplexation of the ^111^In radionuclide from the RIC molecule [37]. Beyond that, it is worth mentioning that the chelating agent influences the biodistribution profile of a RIC. Labile chelators can be cleaved from the antibody molecule by enzymes present in the serum and liver, leading to low-molecular-weight radioactive metabolites that will be eliminated by the kidneys [52].

For the RID and RIT of cancer to be successfully performed, the immunoreactivity of RIC must be preserved, as shown by our immunoreactivity data. Consequently, the specificity to the tumor must also be maintained, which was evidenced by the biodistribution data that showed high accumulation in the SK-BR-3 tumor, as well as great target-to-non-target ratios (tumor/muscle).

However, it is important to mention that high immunoreactivity does not guarantee effective uptake of RICs by tumors. Therefore, there are other issues that can influence this uptake and must be evaluated in its development process, such as the determination of the best molar activity and molar ratio (antibody:chelator) and the lipophilic features [25,53].

Considering the activities of the RICs administered in humans, from 148 to 185 MBq for [^111^In]In-DTPA-trastuzumab and 7400 MBq for [^177^Lu]Lu-DOTA-trastuzumab, the dosimetric data showed that the doses absorbed in the organs for [^177^Lu]Lu-DOTA-trastuzumab are higher than those for [^111^In]In-DTPA-trastuzumab. Absorption is different for the two RICs, since ^177^Lu presented the highest dose in the kidneys, followed by the liver, and ^111^In showed highest dose for the liver, followed by the spleen. Dosimetrically, there is a relative difference in the injected activity between the two radionuclides and also between the energy of both, which can lead to this difference in dose absorption. The fact that the RICs present in some different organs for absorption is due to the affinity of interaction between the organs and the RICs.

Although a greater permanence in the organism is desirable when considering applications in therapy, such permanence must be directly related to the target tissue, in this case, the tumor. In the case of [^177^Lu]Lu-DOTA-trastuzumab, the longer circulation time may represent higher irradiation of non-target organs, especially those that are more vascularized. However, the data presented in this work for both RICs are in accordance with previously published data [54,55].

Taking into account these data, the chelating agents and radionuclides may impair the biological properties of the monoclonal antibodies, such as trastuzumab. However, our data suggest that [^111^In]In-DTPA-trastuzumab and [^177^Lu]Lu-DOTA-trastuzumab are a good theranostic pair for HER2-overexpressing breast tumors. Our data also indicate that SPECT imaging with [^111^In]In-DTPA-trastuzumab should be performed from 72 h to 168 h after RIC administration, and our results are in accordance with previous data [55,56,57]. 

**Figure 8:**
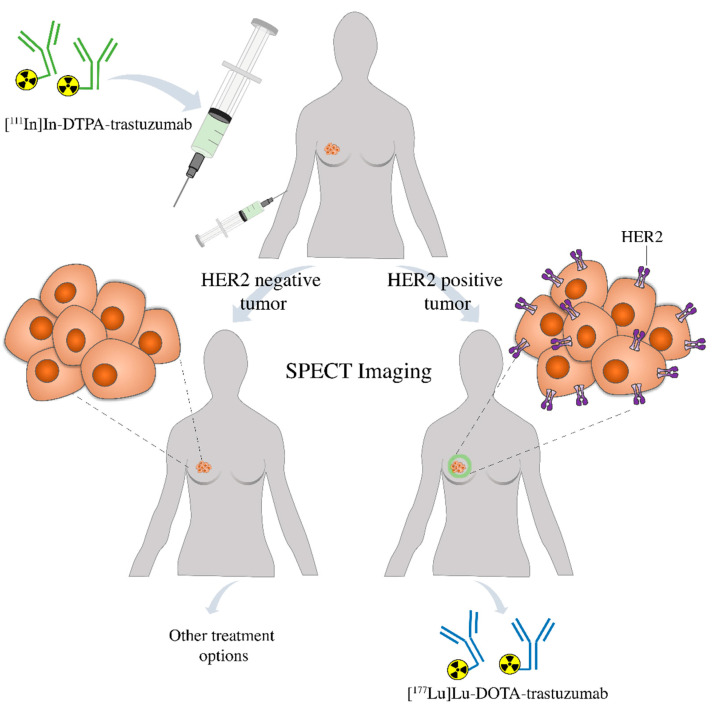
Patient prescreening with [^111^In]In-DTPA-trastuzumab using SPECT imaging prior to therapy allows the identification of individuals positive to HER2 that are more likely to respond to the treatment with [^177^Lu]Lu-DOTA-trastuzumab or be changed to an alternative treatment strategy.

Finally, this comparative study is important in the development of radioimmunotheranostic pairs, which are used for radioimmunodiagnostic and radioimmunotherapy. The diagnostic agent, [^111^In]In-DTPA-trastuzumab, may be used to assess by SPECT imaging the presence of HER2-positive breast tumors in the patient. If positive, [^177^Lu]Lu-DOTA-trastuzumab may be used for target-specific radionuclide therapy. If negative, other treatment options are indicated (Figure 8). Furthermore, in the first case, [^111^In]In-DTPA-trastuzumab may also be used to evaluate the patient’s response to radioimmunotherapy. Further studies should be conducted in patients to confirm the potential of these radioimmunoconjugates as a radioimmunotheranostic pair.

## 5. Conclusions

In conclusion, our results demonstrated that the nature of the chelating agent and radionuclide influences the biological properties of the trastuzumab-based radioimmunoconjugates, highlighting the importance of this evaluation in the development of a theranostic pair. In this work, data showed that the immuno- and radioimmunoconjugation of [^111^In]In-DTPA and [^177^Lu]Lu-DOTA preserved the immunoreactivity of the trastuzumab molecule. Although, different pharmacokinetic, dosimetric and biodistribution behaviors were observed between both RICs, our results suggest that they are suitable for radioimmunotheranostics of HER2 overexpressed-tumors.

## Figures and Tables

**Table 1:** Pharmacokinetic parameters of [^111^In]In-DTPA-trastuzumab and [^177^Lu]Lu-DOTA-trastuzumab determined in normal female BALB/c mice (*n* = 4–5).

Pharmacokinetic Parameters	Symbol	[^111^In]In-DTPA-Trastuzumab	[^177^Lu]Lu-DOTA-Trastuzumab
Fast phase half-life (distribution)	T_½_ Kd	4.43 h	4.64 h
Slow phase half-life (elimination)	T_½_ Ke	774.3 h	1362.0 h
Constant of distribution	Kd	0.16 h^−1^	0.15 h^−1^
Constant of elimination	Ke	0.0008 h^−1^	0.0005 h^−1^
Clearance	CL	0.28 mL·h^−1^	0.04 mL·h^−1^
Volume of distribution	Vd	0.005 L	0.001 L
Effective half-life	T_½_ ef	62.42 h	144.78 h

**Table 2:** Dosimetric data of [^111^In]In-DTPA-trastuzumab and [^177^Lu]Lu-DOTA-trastuzumab, obtained using the biodistribution data (*n* = 6).

Organs	[^111^In]In-DTPA-Trastuzumab	[^177^Lu]Lu-DOTA-Trastuzumab
Red marrow	0.162 mGy/MBq	0.080 mGy/MBq
Kidneys	0.330 mGy/MBq	0.920 mGy/MBq
Liver	0.737 mGy/MBq	0.690 mGy/MBq
Spleen	0.444 mGy/MBq	-

## Data Availability

The data presented in this study are available on request from the corresponding author. The data are not publicly available due to privacy.
